# Low Temperature Geomicrobiology Follows Host Rock Composition Along a Geochemical Gradient in Lau Basin

**DOI:** 10.3389/fmicb.2013.00061

**Published:** 2013-03-27

**Authors:** Jason B. Sylvan, Tiffany Y. Sia, Amanda G. Haddad, Lindsey J. Briscoe, Brandy M. Toner, Peter R. Girguis, Katrina J. Edwards

**Affiliations:** ^1^Department of Biological Sciences, University of Southern CaliforniaLos Angeles, CA, USA; ^2^Department of Earth Sciences, University of Southern CaliforniaLos Angeles, CA, USA; ^3^Department of Soil, Water, and Climate, University of MinnesotaSt. Paul, MN, USA; ^4^Department of Organismal and Evolutionary Biology, Harvard UniversityCambridge, MA, USA

**Keywords:** geomicrobiology, basalt, inactive sulfides, hydrothermal, bacteroidetes

## Abstract

The East Lau Spreading Center (ELSC) and Valu Fa Ridge (VFR) comprise a ridge segment in the southwest Pacific Ocean where rapid transitions in the underlying mantle chemistry manifest themselves as gradients in seafloor rock geochemistry. We studied the geology and microbial diversity of three silicate rock samples and three inactive sulfide chimney samples collected, from north to south, at the vent fields Kilo Moana, ABE, Tui Malila, and Mariner. This is the first study of microbial populations on basaltic andesite, which was sampled at Mariner vent field. Silicate rock geochemistry exhibits clear latitudinal trends that are mirrored by changes in bacterial community composition. α-proteobacteria, ε-proteobacteria, and Bacteroidetes are most common on a silicate collected from Kilo Moana and their proportions decrease linearly on silicates collected further south. Conversely, a silicate from Mariner vent field hosts high proportions of a unique lineage of Chloroflexi unrelated (<90% sequence similarity) to previously recovered environmental clones or isolates, which decrease at ABE and are absent at Kilo Moana. The exteriors of inactive sulfide structures are dominated by lineages of sulfur oxidizing α-proteobacteria, γ-proteobacteria, and ε-proteobacteria, while the interior of one chimney is dominated by putative sulfur-reducing δ-proteobacteria. A comparison of bacterial communities on inactive sulfides from this and previous studies reveals the presence of a clade of uncultured Bacteroidetes exclusive to sulfidic environments, and a high degree of heterogeneity in bacterial community composition from one sulfide structure to another. In light of the heterogeneous nature of bacterial communities observed here and in previous studies of both active and inactive hydrothermal sulfide structures, the presence of numerous niches may be detected on these structures in the future by finer scale sampling and analysis.

## Introduction

The Eastern Lau Spreading Center (ELSC) and Valu Fa Ridge (VFR) comprise the southern portion of the Lau Basin back-arc spreading center, located between the islands of Samoa and Tonga in the southwest Pacific Ocean. Hydrothermal venting was discovered in Lau Basin along the VFR in 1989 (Fouquet et al., 1991) and was subsequently discovered at various other sites along the ELSC and VFR (Langmuir et al., 2004; Ishibashi et al., 2006). The geochemistry of both host rock composition and hydrothermal fluids in Lau Basin changes along the north-south gradient from mid-ocean ridge-like basalt in the north to subduction influenced andesite in the south over the course of <600 km (Escrig et al., 2009; Dunn and Martinez, 2011; Mottl et al., 2011). This is a steeper gradient than seen anywhere else along the global mid-ocean ridge system.

Given the observed gradients in host rock composition and vent fluid geochemistry, Lau Basin is an ideal location to study how geochemistry influences the distribution and composition of biological communities. It has been shown that the gradient in chemistry influences distributions of megafauna (Podowski et al., 2010) and microbes on active sulfide chimneys (Flores et al., 2012), but no work currently exists examining microbiology on the host rock or inactive hydrothermal sulfides. Seafloor-exposed silicates (thus far basalts are the only seafloor silicates sampled) are known to host diverse microbial communities at mid-ocean ridges (Lysnes et al., 2004; Mason et al., 2009; Santelli et al., 2009), but no samples have been analyzed from back-arc systems, where geochemical controls on silicate microbiology can be tested explicitly. Recent work has also illustrated that microbial communities thrive on inactive hydrothermal sulfides long after venting ceases (Rogers et al., 2003; Suzuki et al., 2004; Kato et al., 2010; Sylvan et al., 2012a). A succession occurs on these sulfides whereby the microbial community present on inactive sulfides is different from that on active structures. This is likely due to a change in mineralogy brought on by the drastic decrease in temperature and disappearance of the reduced substrates in hydrothermal fluids once the vent dies. Similar mineralogical controls are present on other low temperature deep-sea deposits, including basalts, where the microbial community appears to be selected by substrate type (Toner et al., 2013); bacterial communities collected from seafloor basalts are more similar to each other than to communities on other substrates. The same is true for bacterial communities on inactive sulfides from the East Pacific Rise (EPR), the Okinawa Trough, and Indian Ocean Ridge (Suzuki et al., 2004; Toner et al., 2013). Both seafloor basalts and inactive hydrothermal sulfide structures can be considered extreme environments due to their high metal content and elevated concentrations of elements considered toxic to most life, such as copper.

We examined the geology and microbiology of low temperature deposits collected from four vent fields along the ELSC and VFR during summer 2009. Specifically, we seek to test the hypothesis that bacterial communities are selected by gradients in host rock composition on silicates [basalts and basaltic andesite, classified using total alkalis versus silica (Le Bas and Streckeisen, 1991)] and to determine if bacterial communities on basaltic andesite, for which no data currently exists, differ from those on basalt. We also seek to further understand bacterial communities on inactive sulfides, for which limited data exists.

## Materials and Methods

### Sample collection

**Figure 1:**
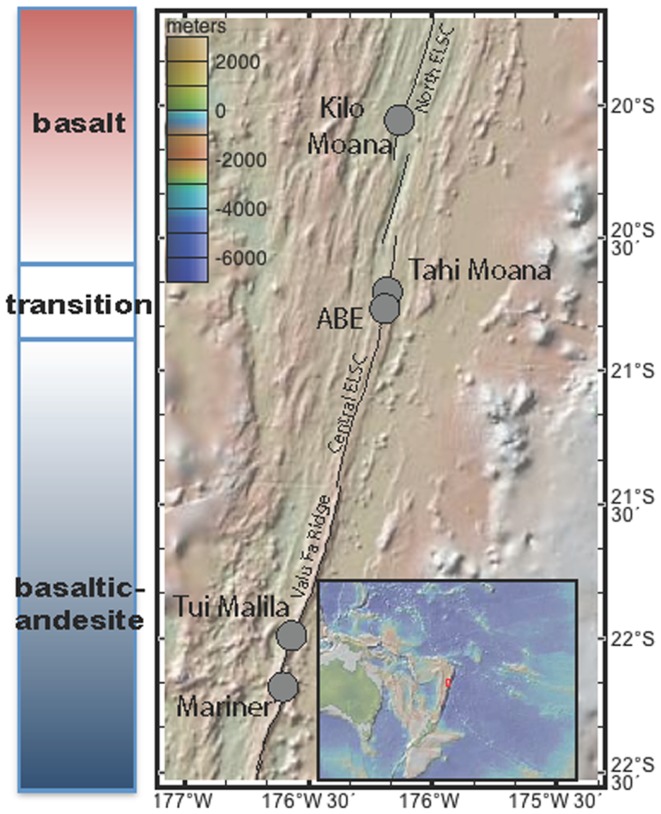
**Map of southern Lau Basin, with the Eastern Lau Spreading Center and Valu Far Ridge**. Samples were collected from Kilo Moana, ABE, Tui Malila, and Mariner vent fields.

**Figure A1:**
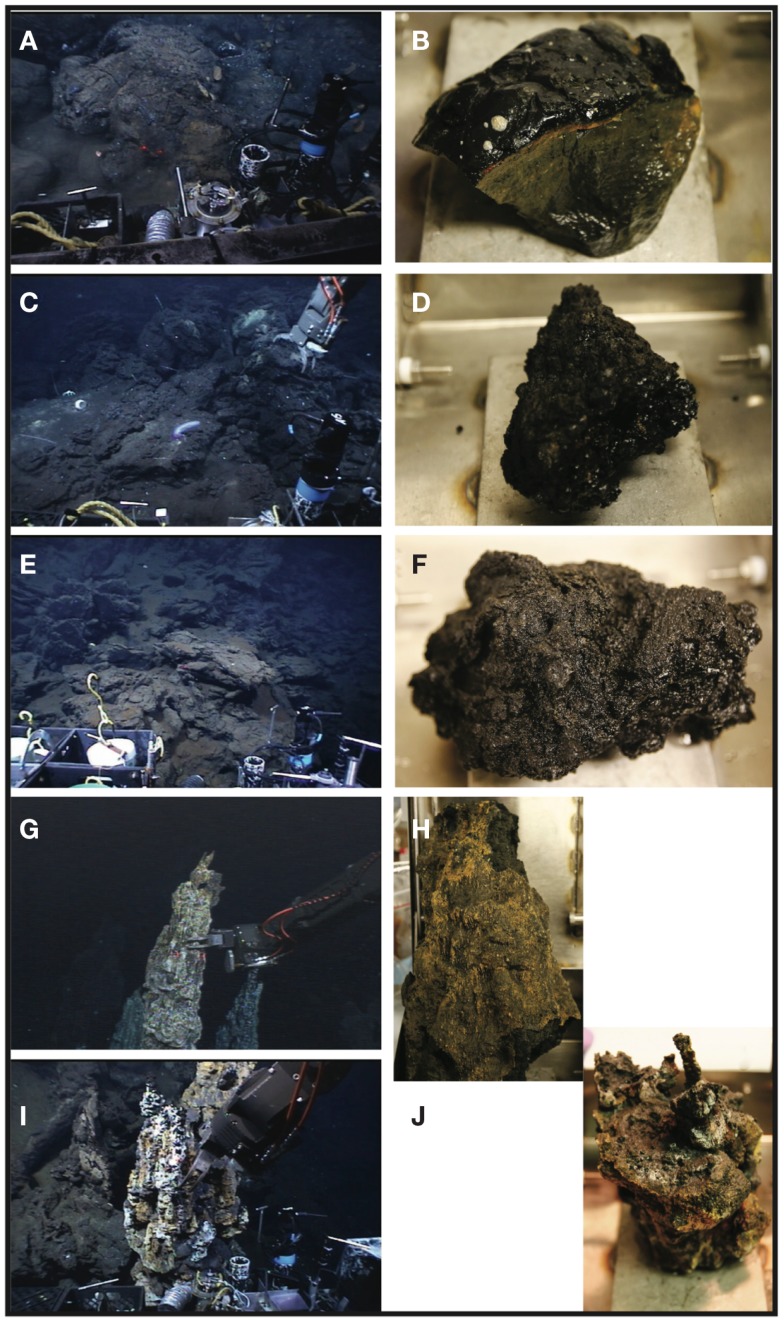
**Seafloor and shipboard photographs of samples collected during this study**. Sample KiMba as it appeared on the seafloor **(A)** and in the sampling box **(B)**. Sample ABEba on the seafloor **(C)** and onboard **(D)**. Sample Marba as it appeared on the seafloor **(E)** and on the ship **(F)**. Sample ABEs as it was collected by *ROV* Jason II **(G)** and on the ship **(H)**. Sample TuiMs, photographed during collection **(I)** and on the ship **(J)**.

Five seafloor rock samples were collected from the ELSC and VFR (Figure 1) during cruise TN-235 on the *R/V Thompson* with *ROV* Jason II during 16 May–08 June 2009. Silicates located outside areas of diffuse flow and sulfides that appeared inactive on the seafloor were collected from four vent fields (Figure A1 in Appendix; Table 1). Samples were collected using *ROV* Jason II and then placed in sealed bioboxes on the sampling tray of the ROV for the remainder of the dive, isolating them in seawater from the collection site for the remainder of the dive. Once on deck, the samples were removed from the bioboxes and immediately processed in flame sterilized steel boxes, where they were separated from the seawater in the bioboxes, with a flame sterilized hammer and chisel. Aliquots of rock chips removed from the larger sample for DNA analysis were placed in 5 mL centrifuge tubes and immediately frozen at −80°C. Subsamples for optical mineralogy (*via* thin section analysis), X-ray diffraction (XRD), and elemental analysis were allowed to air dry on the ship and were subsequently stored at room temperature.

**Table 1:** **Samples descriptions and bacterial biomass estimates**.

Sample	Vent field	Rock type	Rock mineralogy (thin section)	Rock mineralogy (XRD)	Bacterial cells g^−1^ rock* (% Bacteria versus Archaea)	Archaeal cells g^−1^ rock*
KiMba	Kilo Moana	Silicate; basalt	Augite (<5%), Plagioclase (5–10%), Aegirine-augite (<5%), amorphous glass, not porous	Major species: diopside, aegirine-augite Minor species: augite	4.14 ± 0.06 × 10^5^ (93.6%)	2.82 ± 0.69 × 10^4^
ABEba	ABE	Silicate; transitional	Augite (35%), Plagioclase (20%), moderately altered, not porous	Major species: albite, augite Minor species: labradorite	5.20 ± 0.12 × 10^4^ (99.7%)	1.49 ± 0.27 × 10^2^
Marba	Mariner	Silicate; basaltic andesite	Augite (5%), Plagioclase (5–10%), little to no alteration, 40% porous	Signal too amorphous	9.97 ± 1.12 × 10^3^ (15.4%)	5.47 ± 1.86 × 10^4^
ABEsO**	ABE	Outside of an inactive sulfide	Poorly crystalline, pyrite, sphalerite	Sphalerite, pyrite, barite	9.92 ± 0.34 × 10^7^ (100%)	Below detection
ABEsIN	ABE	Inside of an inactive sulfide	nd	nd	1.15 ± 0.04 × 10^7^ (99.8%)	2.87 ± 0.18 × 10^4^
TuiMs	Tui Malila	Inactive sulfide	Cinnabar, pyrite, sphalerite, anhydrite	Barite, sphalerite, pyrite	3.65 ± 0.10 × 10^7^ (100%)	Below detection

*As determined by qPCR, ±Standard Error.

**Mineralogical analysis was completed for the bulk sample for ABEs, but DNA analysis was completed on two separate samples from the same chimney, one from the exterior and one from the interior conduit.

### Thin section analysis

Standard thin sections (30 μm thick) were made from representative portions of each sample by Spectrum Petrographics, Inc. (Vancouver, WA, USA) and were analyzed using a polarizing petrographic microscope (Zeiss AxioImager.M2m with a Zeiss AxioCam HRc camera) with both reflected and transmitted light.

### X-ray diffraction

Approximately 2 cm^3^ of each sample was ground to a powder using mortar and pestle and then mounted on a plastic holder using acetone and a disposable wooden stick to ensure random orientation of the particles. After air drying, samples were analyzed on a Siemens d-500 Diffractometer with a cobalt source. The software program JADE (Materials Data, Inc., v9.3.3) was used for phase identification by peak matching.

### Elemental analysis

Major and minor elemental concentrations were determined from the same subsample used for XRD analysis at the University of Minnesota Analytical Geochemistry Lab. Powders were acid digested prior to analysis. Weight percent of major oxides were determined in triplicate on a Thermo Scientific iCAP 6500 dual view Inductively Coupled Plasma – Optical Emission Spectrometer (ICP-OES). Samples were diluted 40-fold prior to analysis with the addition of a Cs matrix modifier and Y as an internal standard and measured using free aspiration of the sample and integrations of five 10 s replicate readings per measurement. Trace elements were determined in duplicate on a Thermo Scientific XSERIES 2 ICP mass spectrometer with an electrospray ionization PC3 Peltier cooled spray chamber, SC-FAST injection loop, and SC-4 autosampler.

### DNA extraction and analysis

DNA was extracted from ∼4 cm^3^ of sample using a CTAB phenol/chloroform extraction (Ausubel et al., 1999). qPCR for bacteria was carried out as described previously using primers 338f [5′-ACT CCT ACG GGA GGC AGC AG-3′) and 518r (5′-ATT ACC GCG GCT GCT GG-3′ (Einen et al., 2008)]. qPCR for archaea was carried out using primers 806f [5′-ATT AGA TAC CCS BGT AGT-3′ (Takai and Horikoshi, 2000)] and 922r [5′-YCC GGC GTT GAN TCC AAT T-3′ (Delong, 1992)]. For both bacterial and archaeal qPCR, 16S rRNA copy numbers g^−1^ rock were calculated by multiplying the mean copy number detected from triplicate reactions by the dilution factor (total DNA extraction volume divided by template volume in each qPCR reaction) and divided by the weight of the sample from which DNA was extracted, in grams. The cell number per gram rock was determined by assuming 3.9 16S rRNA gene copies per cell for bacteria and 1.8 16S rRNA gene copies per cell for archaea (Einen et al., 2008). The thermal program employed for both bacterial and archaeal qPCR primer sets was: 10 min at 95°C followed by 45 cycles of 30 s at 95°C, 30 s at 55°C and 25 s at 72°C. Negative controls [polymerase chain reaction (PCR) water included as a template] were included for all qPCR runs, and melt curves for all qPCR products were checked to ensure a single PCR product was generated. Reported values in Table 1 were all greater than the negative control and no samples yielded multiple PCR products. The reported qPCR reactions were run in triplicate.

Polymerase chain reaction of the 16S rRNA gene and subsequent cloning and sequencing of the product was carried out according to Sylvan et al. (2012b). Briefly, universal bacterial primers 27F (5′-GAG TTT GAT CCT GGC TCA G-3′) and 1492R (5′-RGY TAC CTT GTT ACG ACT T-3′) were used for PCR. Three reactions were combined and run out on an agarose gel and then excised and extracted using the Zymoclean Gel DNA Recovery Kit (Zymo Research, Irvine, CA, USA). DNA from the extracted PCR band was cloned into the pCR 4 TOPO vector using the TOPO TA Cloning Kit (Invitrogen, Grand Island, NY, USA) and transformants were plated on LB + 100 μg mL^−1^ampicillin according to the manufacturer’s instructions. Clones were sequenced at the Beckman Coulter Genomics center in Danvers, MA, USA.

16S rRNA contigs were generated using Geneious v5.6 (Drummond et al., 2011). Edited near full-length 16S rDNA sequences were classified and checked for chimeras using the Bellerophon tool of Greengenes (DeSantis et al., 2006b). The resulting sequences were aligned using the Greengenes NAST server (DeSantis et al., 2006a) and imported into ARB (Ludwig et al., 2004) for selection of sequences to include in phylogenetic trees. Closely related cultured strains to contigs were identified using the “Named Isolates” option with the BLAST function on the Greengenes website (http://greengenes.lbl.gov/cgi-bin/nph-blast_interface.cgi). If no closely related isolate to a contig existed, the nearest neighbor from the ARB database was identified. Closely related isolates and/or sequences were aligned with sequences from this study using MEGA 5 (Tamura et al., 2011). Phylogenetic trees were constructed following manual adjustment of this alignment using both the neighbor-joining method, based on the maximum composite likelihood model and gamma distribution, and maximum likelihood analysis, based on the Jukes–Cantor model with a Gamma distribution. Both types of phylogenies were tested using 1000 bootstrap replicates. Calculation of rarefaction curves and diversity estimates, as well as comparison between clone libraries and those of other studies, was carried out using the software Mothur (Schloss et al., 2009). Pre-clustering (Huse et al., 2010) and the average neighbor clustering algorithm were used to generate distance matrices from which rarefaction curves, estimates of shared richness (*Jclass*, *Jest*), and estimates of shared structure (theta Yue–Clayton, or Θ*YC*, and Bray–Curtis) were calculated. An operational taxonomic unit (OTU) cutoff of 97% was used for the rarefaction curves and an OTU cutoff of 95% was used for generating a cladogram for inter-sample comparison. In agreement with other prior work (Toner et al., 2013), we found that using a 95% cutoff to build cladograms yielded clearer results than a 97% cutoff. Using a 95% cutoff for OTUs, however, had little impact on the overall number of OTUs and we therefore maintained the commonly accepted value of 97% for OTUs. The cladograms were converted to a circular tree layout in Genious v5.6 (Drummond et al., 2011) and edited in Adobe Illustrator CS6.

DNA sequences generated for this project were deposited in the National Center for Biotechnology Information (NCBI) database under accession numbers KC682512–KC682862. For a few highly represented sequences, where multiple nearly identical clones were generated from a single sample, only one clone was deposited. They are listed here, followed by the number of additional clones they represent: ABEsO_A7 (25 additional clones), Marba_A2 (09 additional clones), ABEsIN_H1 (15 additional clones), ABEsIN_A1 (47 additional clones), and TuiMs_A6 (24 additional clones).

## Results

### Sample descriptions, silicates

**Figure 2:**
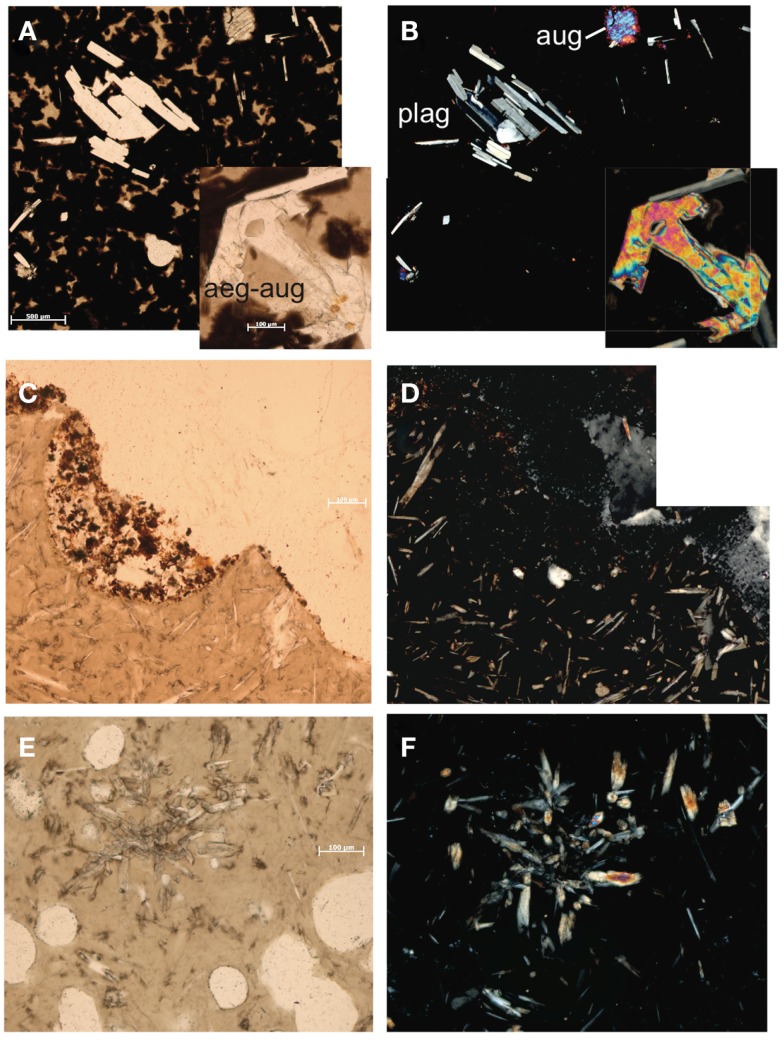
**Thin section photomicrographs of silicate samples**. Plain polar light photomicrograph of basalt KiMba **(A)** and the same view in crossed polars **(B)**. Plain polar light photomicrograph of basalt ABEba **(C)** and crossed polars of the same view **(D)**. Plain polar light photomicrograph of sample basaltic andesite Marba **(E)** and crossed polar image of the same view **(F)**.

Sample KiMba is a basalt collected from Kilo Moana vent field with a visible glassy rim underlain by ground mass. We sampled the glassy rim. The thin section revealed that KiMba is porphyritic with large phenocrysts in an amorphous glassy matrix that are encroached by spherules. KiMba is not porous, and plagioclase [(Na,Ca)(Si,Al)_4_O_8_], aegirine-augite [(Ca,Na)(Mg,Fe^2+^,Fe^3+^)(Si_2_O_6_)], and augite [(Ca,Na)(Mg,Fe,Al,Ti)(Si,Al)_2_O_6_] are represented in the thin section (Figure 2). XRD analysis revealed the presence of diopside [CaMg(Si_2_O_6_)] in this sample (Table 1). Sample ABEba is a basalt collected from ABE vent field with an oxidized rim. It was not porous and is rich in augite and plagioclase (Figure 2). The XRD pattern revealed the presence of albite (NaAlSi_3_O_8_), augite and labradorite [(Na,Ca)(Si,Al)_4_O_8_]. Sample Marba is basaltic andesite collected from Mariner vent field. It is very porous (Figure 2) with little to no alteration. Plagioclase and augite were detected by thin section analysis, but the XRD signal was too weak to identify specific minerals.

### Inactive sulfides

**Figure 3:**
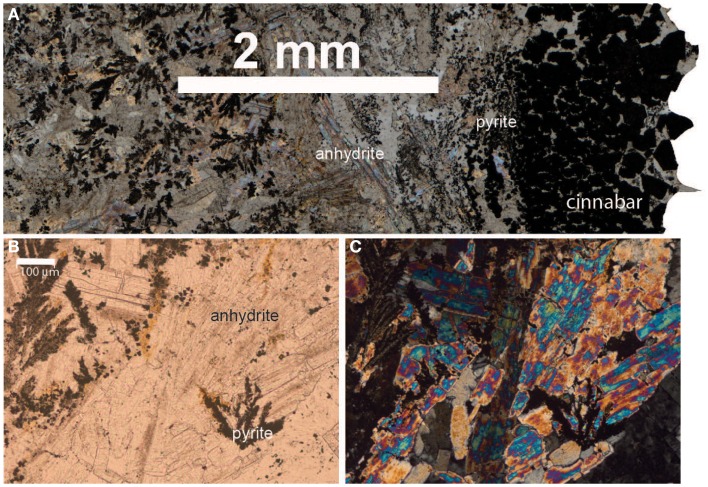
**Thin section photomicrographs of inactive sulfide sample TuiMs**. Plain polar light photomosaic shows transition from cinnabar and pyrite on the rim to anhydrite and pyrite **(A)**. A close up in plain polar light of the anhydrite and pyrite **(B)** and the same view in crossed polars **(C)**.

Sample ABEs was broken off an inactive sulfide chimney located in ABE vent field. It is poorly crystalline and slightly oxidized. Light microscopy of samples in thin section revealed the presence of pyrite (FeS_2_) and sphalerite (ZnS; data not shown); and barite (BaSO_4_) was identified in the XRD pattern. A portion of this sample was collected from the outside wall of the chimney (ABEsO) and a separate section was sampled from the inside (ABEsIN) for analysis of the microbial community. Sample TuiMs is a zoned chimney that appeared inactive (no venting or shimmering fluid observed exiting the chimney) on the seafloor at the time of sampling from the Tui Malila vent field. Sulfide minerals are present on the outer rim of the thin section and sulfide and sulfate minerals are present away from the outer rim (Figure 3). On the outer tannish red to deep maroon rim, euhedral cinnabar (HgS) coats and encrusts anhedral pyrite. The interior edge of the thin section is characterized by anhydrite (CaSO_4_), sphalerite, and minor anhedral pyrite. XRD analysis reveals the presence of barite, sphalerite, and pyrite.

### Geochemistry

Elemental analysis confirms transitions in host rock composition from north to south in the ELSC. Weight percent of Ba, K, P, Si, and Sr oxides and concentrations of V, Cu, Rb, Sr, Ba, La, Ce, Pr, Nd, and Th all increase from north to south in the silicates (Tables 2 and 3). Concentrations of Sc and Co follow the opposite trend. Based on analysis of total alkalis versus silica, KiMba, and ABEba are both sub-alkaline basalts while Marba is sub-alkaline basaltic andesite (data not shown).

**Table 2:** **Major oxide composition of the rocks sampled**.

Oxide	KiMba	ABEba	Marba	ABEs	TuiMs
Al_2_O_3_	14.76	14.5867	15.1733	0.1282	0.15713
BaO	0.0008	0.0148	0.0203	1.8943	3.175
CaO	12.0467	8.423	9.1697	0.2079	1.7117
Fe_2_O_3_	11.22	12.9633	11.9967	11.9	2.8467
K_2_O	0.0366	0.2393	0.2588	0.0455	0.0514
MgO	7.7087	4.098	4.7983	0.0414	0.047
MnO	0.1839	0.3112	0.208	0.1575	0.0134
Na_2_O	2.106	3.085	2.9113	0.4025	0.1844
P_2_O_5_	0.0787	0.1402	0.15	0.0179	0.0025
SiO_2_	50.88	51.8933	52.5033	8.1507	8.1913
SrO	0.0088	0.0167	0.0183	0.3631	0.2826
TiO_2_	1.0097	1.389	1.3247	0.0105	0.0086
ZrO_2_	0.0115	0.0155	0.0139	0.0015	0.0017

*Note that totals for ABEs and TuiMs are not 100%; the remaining weight percent is composed primarily of barite for these two sulfides*.

**Table 3:** **Trace element compositions of the rocks sampled**.

Element	KiMba	ABEba	Marba	ABEs	TuiMs
^7^Li	5.13	7.51	6.14	0.74	0.33
^31^P	271.90	504.30	535.05	70.32	18.96
^45^Sc	43.61	36.56	35.80	0.03	0.04
^51^V	340.50	417.55	428.85	6.98	3.61
^52^Cr	175.50	2.45	28.76	0.89	0
^55^Mn	1500.50	2821.50	1685.50	1277.50	110.50
^59^Co	42.91	36.95	33.30	0.05	0.01
^60^Ni	69.31	23.89	23.84	3.04	0.48
^65^Cu	77.42	77.74	80.19	2995.00	1950.00
^66^Zn	86.48	133.50	84.49	102150.0	68325.0
^71^Ga	15.66	18.13	17.66	2.82	11.93
^72^Ge	2.49	2.73	2.63	33.32	16.25
^85^Rb	0.74	3.96	5.19	1.70	1.08
^86^Sr	75.41	143.00	155.65	3014.00	2267.00
^89^Y	25.83	32.86	29.99	0	0
^91^Zr	55.06	83.52	74.71	1.92	0.34
^93^Nb	0.55	1.01	0.78	0	0
^95^Mo	0	1.39	0.46	50.80	15.08
^111^Cd	0.07	0.17	0.07	96.67	196.80
^118^Sn	0.52	0.81	0.65	0	0
^121^Sb	0	0	0	42.26	68.94
^133^Cs	0	0	0	0.11	0
^137^Ba	7.73	143.05	195.60	662.55	932.90
^139^La	1.62	3.09	3.61	0.67	0.47
^140^Ce	5.69	9.31	10.38	1.02	0.56
^141^Pr	1.04	1.67	1.73	0.09	0.04
^146^Nd	6.33	9.64	9.71	0.24	0.10
^147^Sm	2.45	3.37	3.35	0.02	0.00
^151^Eu	0.92	1.24	1.21	0.07	0.06
^157^Gd	3.63	4.81	4.65	0.01	0.01
^159^Tb	0.68	0.86	0.82	0.00	0.00
^163^Dy	4.93	6.14	5.82	0	0
^165^Ho	1.07	1.31	1.23	0.00	0.00
^166^Er	3.30	4.04	3.75	0.00	0
^169^Tm	0.48	0.59	0.55	0.00	0.00
^172^Yb	3.23	3.94	3.65	0.00	0
^175^Lu	0.50	0.61	0.56	0.00	0.00
^178^Hf	1.76	2.49	2.40	0.02	0
^181^Ta	0	0	0	0	0.01
^182^W	0.07	0.12	0.07	0.05	0.00
^208^Pb	0	3.07	1.41	3749.00	4972.50
^232^Th	0.06	0.16	0.30	0.00	0
^238^U	0	0	0	1.05	0.00

*All concentrations are in ppm*.

Inactive sulfides ABEs and TuiMs were much more elevated in Ba, Cu, Zn, and Sr than the silicate samples (Tables 2 and 3). Sulfide TuiMs had nearly 10-fold more weight percent Ca than ABEs. A significant portion of ABEs and TuiMs was not acid digestible after 2 weeks in acid. This portion was collected and analyzed by XRD, which revealed that is composed of barite for both ABEs and TuiMs. With the exception of Sc and Ba, sample ABEs had higher trace element concentrations than sample TuiMs.

### Microbiology

Bacterial and archaeal biomass were estimated via qPCR on all samples, including one sample each collected from the outside and inside of ABEs (ABEsO and ABEsIN, respectively). Bacteria accounted for >90% of the combined Bacteria + Archaea on all samples except for Marba and bacterial biomass was higher on the inactive sulfide samples (7 × 10^6^–6 × 10^7^ cells g^−1^) than on the silicates (∼1 × 10^4^–6 × 10^5^ cells g^−1^; Table 1). It must be noted, however, that because the primers used in qPCR are likely biased, this method provides an estimate, and the derived proportions are also estimates.

**Figure 4:**
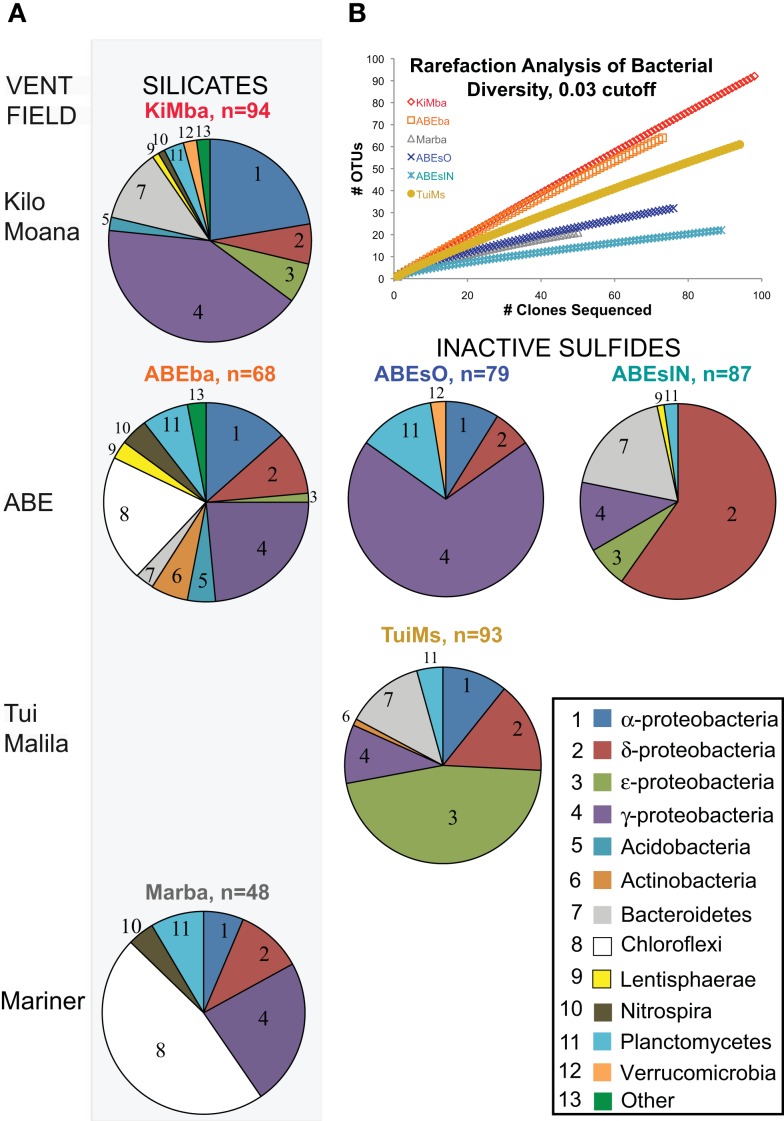
**(A)** Bacterial distributions recovered from samples in this study. Samples are arranged in rows from north to south in Lau Basin and the silicate samples are in the left column of pie charts while the inactive sulfides are to the left. The number of clones recovered per sample is indicated next to the sample name. Bacterial phyla/class are indicated by the numbers in each pie slice, according to the key at bottom right. **(B)** Rarefaction analysis of clone libraries from this study. Clones were aligned, pre-clustered (Huse et al., 2010), and analyzed at the 97% similarity level using the average neighbor method in the software mothur (Schloss et al., 2009).

Given the high proportion of Bacteria versus Archaea, and their presence on all samples, we chose to analyze bacterial diversity. We PCR amplified and cloned near full-length bacterial 16S rRNA from all six samples; 469 total clones were recovered and sequenced, with 48–93 clones per sample (Figure 4). Rarefaction analysis using a 97% cutoff for OTU designation reveals that both basalt samples are more diverse than the other samples (Figure 4). Sample Marba exhibits the lowest diversity.

**Figure A2:**
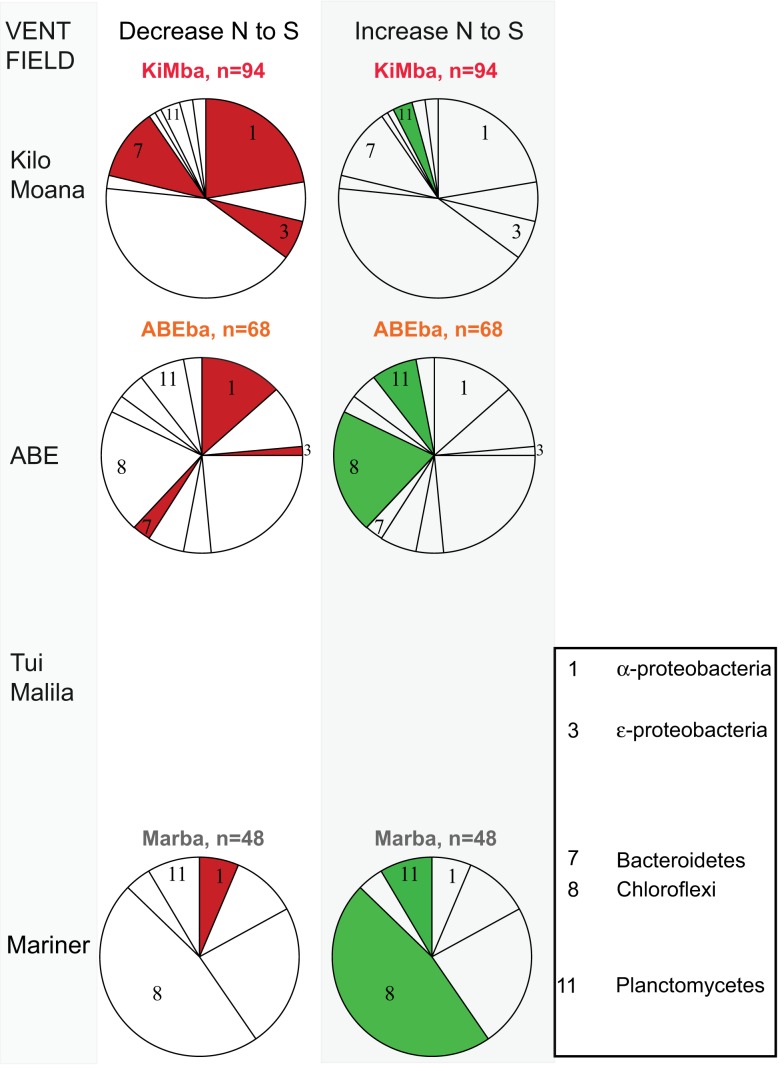
**Bacterial distributions of phyla/classes that either decrease (left column) or increase (right column) in proportionality from north (Kilo Moana) to south (Mariner) on silicate samples in this study**. Key is the same as Figure 4, except that phyla/classes that decrease from north to south are all red, those that increase from north to south are all green, and those that do not show a north to south pattern are not included.

Further analyses of bacterial OTUs recovered at the phylum level reveals that some phyla exhibit north to south patterns in distribution on the silicates (Figure 4; Figure A2 in Appendix). α-Proteobacteria decrease in proportion from north to south, as do ε-proteobacteria and Bacteroidetes. In contrast, Planctomycetes and Chloroflexi increase in proportion from north to south. Because there are only two inactive sulfides, we cannot delineate any conclusions about north to south distributions of bacterial communities on hydrothermally inactive sulfides.

Bacterial populations on the inactive sulfide samples are markedly different from those on the silicates at the phylum level, and each of the three inactive sulfide samples are different from each other. No Chloroflexi were recovered from the sulfides, but given the smaller datasets generated by clone libraries compared to pyrosequencing (e.g., Flores et al., 2012), the absence of taxa must be treated with caution. Sample ABEsO is dominated by γ-proteobacteria, but the inner conduit of the same chimney, ABEsIN, is dominated by δ-proteobacteria. Some ε-proteobacteria and Bacteroidetes are also recovered from ABEsIN, but not from ABEsO. Sample TuiMs harbors a high proportion of Proteobacteria in general, and ε-proteobacteria are the most commonly recovered class. δ-Proteobacteria, γ-proteobacteria, and Planctomycetes are the only phyla recovered from all six samples.

**Figure 5:**
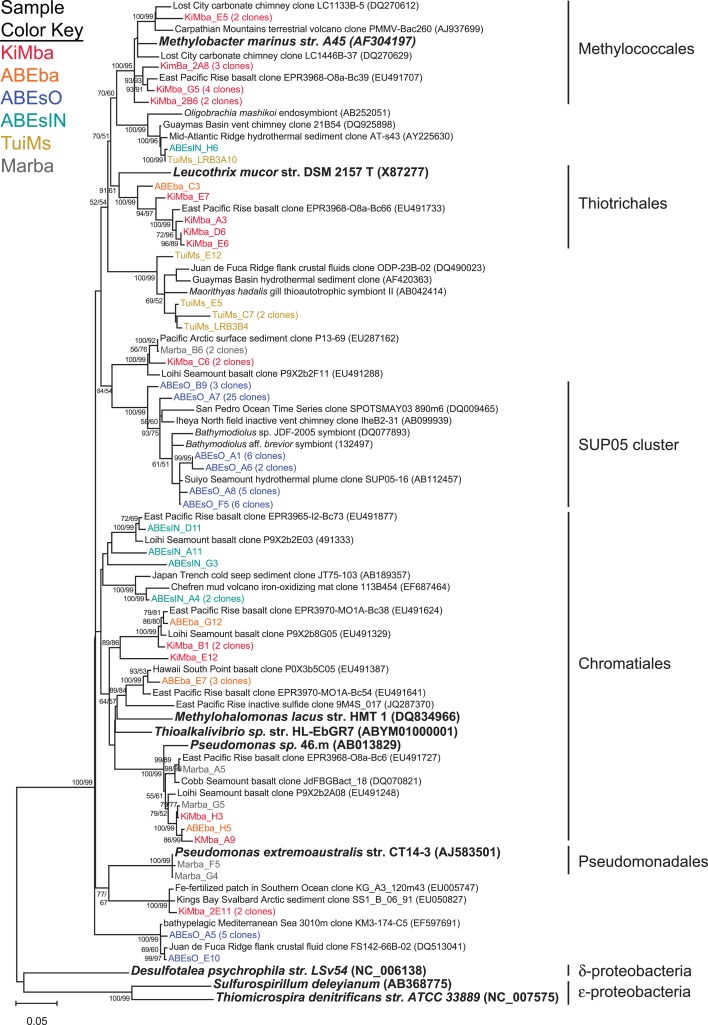
**Phylogenetic tree of representative clones from this study that fall within the γ-proteobacteria**. Clones with more than one representative, or for which multiple members fell into a single clade, were chosen. Numbers in parentheses after some clones indicate total number of clones represented by the single clone depicted. The tree was generated in MEGA 5 using the maximum likelihood method with the Jukes–Cantor model and a Gamma distribution and 1000 bootstrap replicates. The same alignment was used to generate a neighbor-joining tree with the maximum composite likelihood method and 1000 bootstrap replicates. Nodes where both methods agree and bootstrap support was >50% are indicated with bootstrap values from the neighbor-joining tree on the left and the maximum likelihood tree on the right. Samples are color-coded and no representative clones are shared across multiple samples. *D. psychrophila*, *S. deleyianum*, and *T. denitrificans* were used as outgroups.

Recovered clones that group within the γ-proteobacteria include members of the orders Methylococcales, Thiotrichales, Chromatiales, and Pseudomonadales (Figure 5). Clones classified as Methylococcales are recovered only from sample KiMba. Those within the Thiotrichales are from the two basalt samples, while clones that fall within the Chromatiales order were recovered from all three silicate samples. Forty-seven clones recovered from ABEsO fall within the SUP05 clade of γ-proteobacteria, but these are exclusive to this sample. Nearly all of the clones that fall within the γ-proteobacteria are most similar to clones or isolates from other mid-ocean ridge or sedimentary settings.

**Figure 6:**
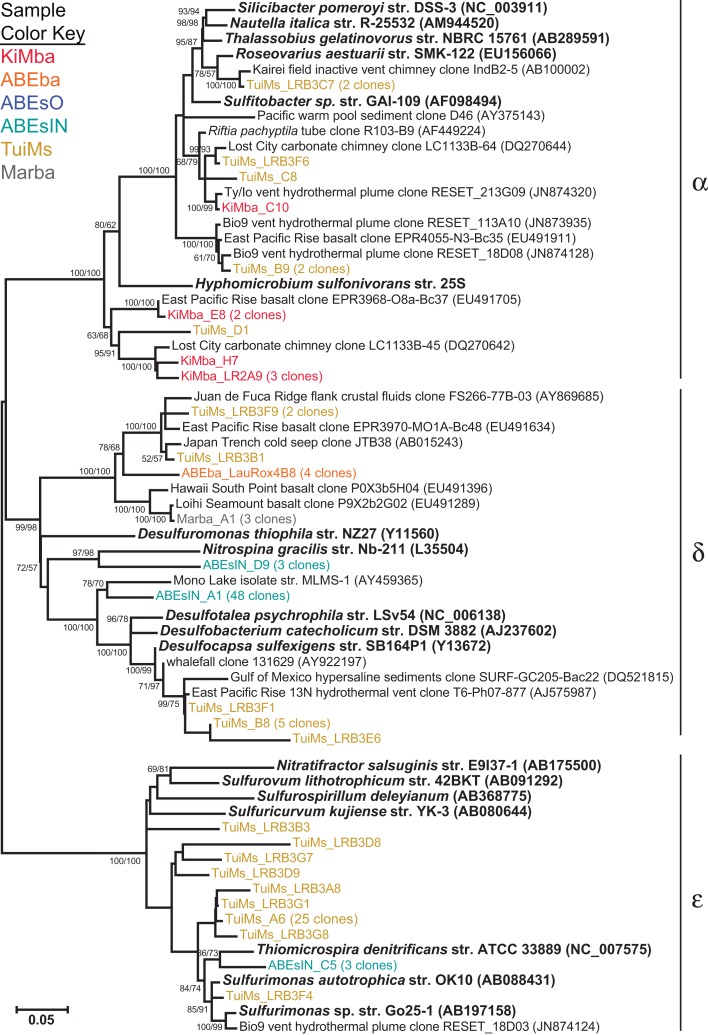
**Phylogenetic tree of representative clones from this study that fall within the α-, δ-, and ε-proteobacteria**. Clones with more than one representative, or for which multiple members fell into a single clade, were chosen. Numbers in parentheses after some clones indicate total number of clones represented by the single clone depicted. The tree was generated in MEGA 5 using the maximum likelihood method with the Jukes–Cantor model and a Gamma distribution and 1000 bootstrap replicates. The same alignment was used to generate a neighbor-joining tree with the maximum composite likelihood method and 1000 bootstrap replicates. Nodes where both methods agree and bootstrap support was >50% are indicated with bootstrap values from the neighbor-joining tree on the left and the maximum likelihood tree on the right. Samples are color-coded and no representative clones are shared across multiple samples.

Some of the clones from samples KiMba and TuiMs are closely related to α-proteobacteria within the Roseobacter clade and the genus *Hyphomicrobium* (Figure 6). Clones that fall within the δ-proteobacteria are related to *Nitrospina* and *Desulfocapsa* and uncultured clones from other hydrothermal vent and sedimentary environments. Thirty-three clones recovered from TuiMs and three from ABEsIN are most similar to isolates in the genus *Sulfurimonas* [*Thiomicrospira denitrificans* was recently reclassified into this genus (Takai et al., 2006)].

**Figure 7:**
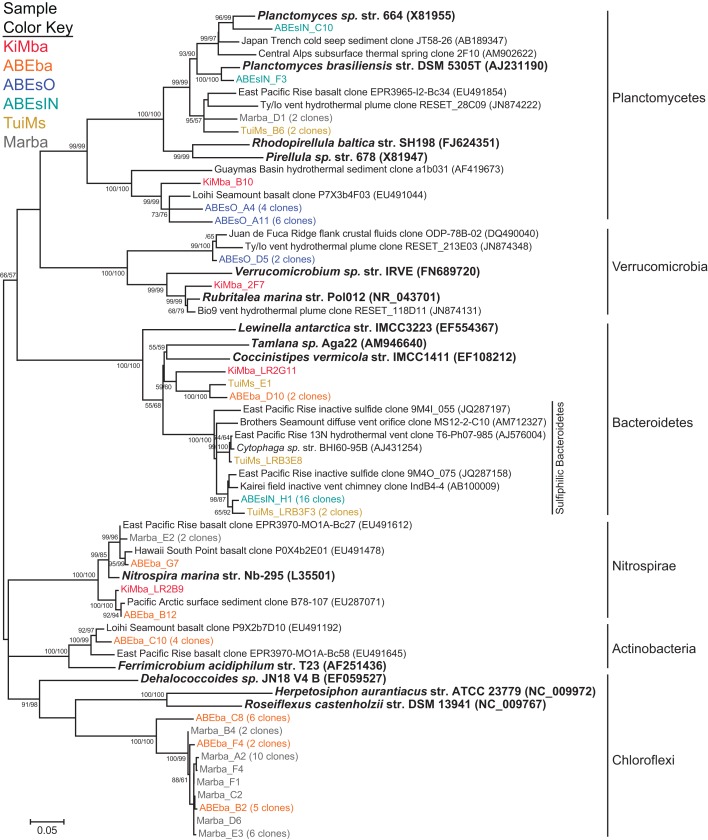
**Phylogenetic tree of representative clones from this study that fall within the non-proteobacterial phyla**. Clones with more than one representative, or for which multiple members fell into a single clade, were chosen. Numbers in parentheses after some clones indicate total number of clones represented by the single clone depicted. The tree was generated in MEGA 5 using the maximum likelihood method with the Jukes-Cantor model and a Gamma distribution and 1000 bootstrap replicates. The same alignment was used to generate a neighbor-joining tree with the maximum composite likelihood method and 1000 bootstrap replicates. Nodes where both methods agree and bootstrap support was >50% are indicated with bootstrap values from the neighbor-joining tree on the left and the maximum likelihood tree on the right. Samples are color-coded and no representative clones are shared across multiple samples.

A diverse group of clones were recovered from our samples that group with phyla outside the Proteobacteria (Figure 7). Among these are a clade of Chloroflexi recovered from the two silicates ABEba and Marba. None of these clones are closely related to any cultured isolates, and the most similar environmental clone in the NCBI database is only 95% similar to clone ABEba_C8. Eighteen clones recovered from only inactive sulfides TuiMs and ABEsIN, represented by clones ABEsIN_H1 and TuiMs_LRBF3, group within a cluster of Bacteroidetes clones recovered from other inactive sulfides. This cluster falls within a larger clade, to which clone TuiMs_LR3E8 belongs, comprised of Bacteroidetes clones recovered exclusively from sulfidic environments.

Nitrospirae were recovered from the three silicates, but not from the sulfides. These clones group most closely with *Nitrospira marina* (Figure 7). Four clones represented by clone ABEba_C10 are 85% similar to *Ferrimicrobium acidiphilum* str. T23 and *Acidimicrobium ferrooxidans*, both iron oxidizing acidophiles.

### Correlations between geochemistry and microbiology

We used the elemental composition and phylogenetic data to determine correlations between geochemistry and microbiology. For this purpose, the same elemental data was used for ABEsO and ABEsIN because a bulk sample from this sulfide was analyzed for geochemistry. Ideally, one would compare the presence of individual OTUs in a sample with geochemical data to identify correlations between the two. However, using a 97% similarity cutoff for OTUs, only one OTU, comprised by clones TuiMs_LRB3A10 and ABEsIN_H6 (Figure 5), has membership from two different samples, and therefore no correlations exist at this cutoff between OTUs and geochemistry. Using a 10% cutoff only resulted in six OTUs with membership from multiple samples. Therefore, we measured correlations between the geochemical data and the abundance of bacterial phyla, as represented by their percentage on each sample. We do not report correlations between elemental variables alone because larger datasets are available for such purposes (e.g., Escrig et al., 2009).

Nitrospirae and Chloroflexi are the only phyla with significant correlations to geochemistry. Both are strongly positively correlated to P, V, La, Ce, Pr, Nd, Th, P_2_O_5_, and SiO_2_ (Table 4). Nitrospirae are additionally positively correlated to the abundance of Y, Nb, Sn, Tb, Dy, Ho, Er, Tm, Yb, Lu, Li, La, Mn, Ga, Zr, Sm, Eu, Gd, Hf, W, MnO, Na_2_O, TiO_2_, and ZrO_2_, and negatively correlated to Sb. A negative correlation exists between abundance of γ-proteobacteria and δ-proteobacteria, Planctomycetes and Bacteroidetes, Planctomycetes and ε-proteobacteria, and Verrucomicrobia and δ-proteobacteria. There was a positive correlation between the proportion of Bacteroidetes and ε-proteobacteria, Nitrospirae and Chloroflexi, and Verrucomicrobia and γ-proteobacteria.

**Table 4:** **Kendall’s τ correlations between elemental data and bacterial distributions**.

Variable 1	By variable 2	Kendall τ	Prob > | τ|
γ-proteobacteria	δ-Proteobacteria	−0.9429	0.0048
Nitrospirae	Y, Nb, Sn, Tb, Dy, Ho, Er, Tm, Yb & Lu	1	<0.0001
Nitrospirae	Li, Mn, Ga, Zr, Sm, Eu, Gd, Hf, W, MnO, Na_2_O, TiO_2_ & ZrO_2_	0.9549	0.0030
Nitrospirae	P, V, La, Ce, Pr, Nd, Th, P_2_O_5_ & SiO_2_	0.8933	0.0165
Nitrospirae	Sb	−0.8854	0.0190
Nitrospirae	Chloroflexi	0.8262	0.0427
Bacteroidetes	ε-Proteobacteria	0.9412	0.0051
Planctomycetes	Bacteroidetes	−0.9276	0.0077
Planctomycetes	ε-Proteobacteria	−0.8117	0.0499
Chloroflexi	P, V, Rb, La, Ce, Pr, Nd, Th, K_2_O, P_2_O_5_, SiO_2_	0.8575	0.0291
Verrucomicrobia	δ-Proteobacteria	−0.8452	0.0341
Verrucomicrobia	γ-proteobacteria	0.8452	0.0341

*Correlations between pairs of variables that are both elemental data are not included here. Only correlations for which *p* < 0.05 are presented here. When multiple variables are listed in the “by Variable 2” column, this means that both Kendall τ and Prob > | τ| values are the same between Variable 1 and all variables listed in “by Variable 2” column*.

### Inactive sulfide biogeography

**Figure 8:**
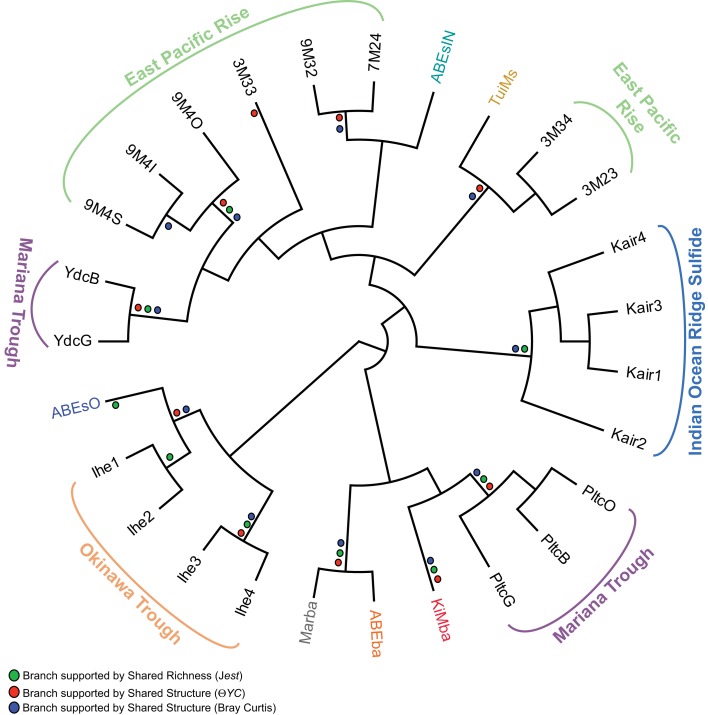
**Cladogram of OTU-based comparison of bacterial communities sampled from inactive sulfides in this and prior studies using the J_class_ algorithm**. The three silicate samples from this study are included. Where the J_class_ analysis agreed with community comparison using Jest, Θ*YC*, and/or Bray–Curtis algorithms, a dot for the agreeing methods is placed at the node in the cladogram. Overall agreement is strong between all four methods save for sample 3M33 from the EPR. Sampling location is indicated for samples from prior studies: EPR (Sylvan et al., 2012a), Mariana Trough (Kato et al., 2010), and the Indian Ocean Ridge and Okinawa Trough (Suzuki et al., 2004). Sample color code for samples from this study is the same as in Figures 4–7.

We compared the bacterial communities on inactive sulfides recovered during this study with those from previous studies (Suzuki et al., 2004; Kato et al., 2010; Sylvan et al., 2012a; Toner et al., 2013) using an OTU-based approach to determine if the bacterial communities on ABEs and TuiMs are similar to that from any previously analyzed inactive sulfides, and also to determine if any biogeographical patterns could be detected amongst bacterial communities on inactive sulfides (Figure 8). The three silicates from the present study were also included in this analysis. Shared richness of the bacterial communities, as determined using the J_class_ and J_est_ calculators, and shared structure as determined using the Yue–Clayton and Bray–Curtis calculators, generated similar results. Sulfide sample TuiMs grouped closely with sulfide rubble collected near K vent at 9°N EPR (3M34 and 3M23), from which a larger proportion of ε-proteobacteria were recovered than other inactive sulfides. ABEsIN grouped with EPR samples from which δ-proteobacteria are prevalent and ABEsO grouped with samples from an inactive sulfide collected from the Okinawa Trough. All three silicates from this study grouped with three samples analyzed from the same inactive sulfide collected from the Mariana Trough.

## Discussion

### Novelty of work

Here we investigated microbiology along a geochemical gradient in the ELSC. This back-arc spreading system is of particular interest because it displays clear trends in host rock geochemistry (Escrig et al., 2009; Dunn and Martinez, 2011; Mottl et al., 2011) that are hypothesized to influence microbial communities. We targeted low temperature deposits from four fields along this geochemical gradient and were able to detect patterns in bacterial community composition that correlate with changes in geochemistry. This is also the first study of microbial populations on basaltic andesite. Our sample set is admittedly small compared to pyrosequencing datasets generated by next generation sequencing methods, but the data recovered are informative and we view this work as an initial survey of low temperature geomicrobiology in Lau Basin. It should also be noted that it is unknown if the organisms detected in this study are active, and that DNA may last for some time in the environment, as indicated by the presence of extracellular DNA in deep-sea sediments (Dell’Anno and Danovaro, 2005).

### Silicates

Our geochemical and mineralogical analysis of the silicates collected reinforces north-south along axis patterns seen for Si, Sr, Ba, La, Ce, Nd, and Th in previous work that looked at fresh lava flows in the ELSC (Escrig et al., 2009; Dunn and Martinez, 2011). It must be pointed out that our samples were not specifically selected for lack of alteration, unlike those in these aforementioned studies. Therefore, deviations from the previous study such as the Ba/Th and Th/La ratios, may be due to oxidative alteration of the collected rocks, or incomplete digestion of Ba-bearing minerals. Accordingly, we use the mineralogical and geochemical data here to better understand the nature of relationships among geochemistry and microbial populations, not to draw larger conclusions about geochemistry in the ELSC.

Bacterial communities on basalts from Lau Basin were more diverse than those on inactive sulfides from this study (Figure 4), in agreement with prior work indicating basalts host extremely diverse bacterial populations (Santelli et al., 2008). Within the sulfide samples, TuiMs is more diverse than both ABEsO and ABEsIN. Inactive sulfide ABEs is ∼150% more enriched in Zn than is TuiMs. A recent comparison of inactive sulfides from the EPR found that Zn-rich chimneys harbor fewer OTUs than Fe-rich sulfides (Toner et al., 2013). This is also true here.

North to south patterns in bacterial community composition occur at the phylum level in the ELSC. Kilo Moana (north) is analogous to a mid-ocean ridge vent field, whereas Mariner (south) is strongly influenced by subduction, and ABE exhibits transitional characteristics between the two end-members. As such, patterns of bacterial membership on the silicate rocks follow this pattern linearly for the α-proteobacteria, ε-proteobacteria, Bacteroidetes, Planctomycetes, and Chloroflexi, where the proportional representation of each phylum on sample ABEba falls between that on samples KiMba and Marba. This is supported in part by the high positive correlation between Bacteroidetes and ε-proteobacteria (both decreased from north to south) and the negative correlation between Planctomycetes and Bacteroidetes (opposite north to south patterns). These gradients are likely drivers for absence of shared OTUs between bacterial communities detected on each sample. This is reinforced by Libshuff analysis, which reveals that each silicate harbors a unique bacterial community. Further, when the inactive sulfides are included in this analysis, all six samples in this study are significantly different from each other.

The bacterial community on sample Marba is quite different from what has been observed on previously studied seafloor-exposed basalts (Lysnes et al., 2004; Mason et al., 2009; Santelli et al., 2009), and also from the other basalt samples in this study. The qPCR results indicate that biomass is very low on this sample compared to mid-ocean ridge basalts, which can host bacterial biomass up to 10^9^ cells g^−1^(Santelli et al., 2008). Archaea outnumbered Bacteria on sample Marba, whereas all previously measured basalt samples had higher bacterial populations by ∼9:1 (Einen et al., 2008; Santelli et al., 2008), as did samples KiMba and ABEba.

The high proportion on Marba of clones that fall within the phylum Chloroflexi is also unusual compared to previously analyzed basalts; while this phylum is often represented as a few percent of the total clones on seafloor-exposed basalt samples from mid-ocean ridge settings, the large community membership of a monophyletic clade of Chloroflexi, as seen here, has not previously been observed. Clones from the same clade were also recovered from sample ABEba and all members of this clade are <90% similar to the most closely related sequences in the NCBI database. As such, it is impossible to speculate on the ecological niche for the organisms represented by these clones, or whether their presence is or is not affected by substrate geochemistry. The Chloroflexi clones recovered from samples Marba and ABEba fall within the class Anaerolineae, which was also recovered from active hydrothermal vent sulfides at Mariner (Takai et al., 2008; Flores et al., 2012). However, these studies found low proportions of Anaerolineae, and the clones from active chimneys are distantly related to the clones recovered from samples Marba and ABEba. The classification of the Chloroflexi clones from Marba and ABEba within the same class as those found on active vents indicates that, while unusual, these are not resultant from contamination.

Our finding of a microbial community on a silicate from Mariner vent field that is distinct from those collected at ABE and Kilo Moana mirrors a detailed study of bacterial and archaeal diversity on active sulfide chimneys in the ELSC, which also found the microbial communities at Mariner to be distinct (Flores et al., 2012). In that study, the unique geochemistry of the hydrothermal fluids was a driver of the microbial community structure; we believe that the geochemistry of the host rock silicates at Mariner has an equal influence on the microbial communities they support.

Clones classified within the phylum Nitrospirae and the genus *Nitrospira* and the γ-proteobacterial order Chromatiales were recovered from all three silicate samples. The clones related to *Nitrospira* fall within *Nitrospira* sublineage IV, which are known to oxidize nitrite to nitrate mixotrophically (Daims et al., 2001). Members of the Chromatiales are chemolithotrophic sulfur oxidizers that may be capable of autotrophy (Brenner et al., 2005). Clones in both these phyla were closely related to environmental clones recovered from seafloor-exposed basalts from the Juan de Fuca Ridge, Loihi Seamount, and the EPR (Mason et al., 2009; Santelli et al., 2009), indicating that these are widespread lineages on seafloor basalts.

**Figure A3:**
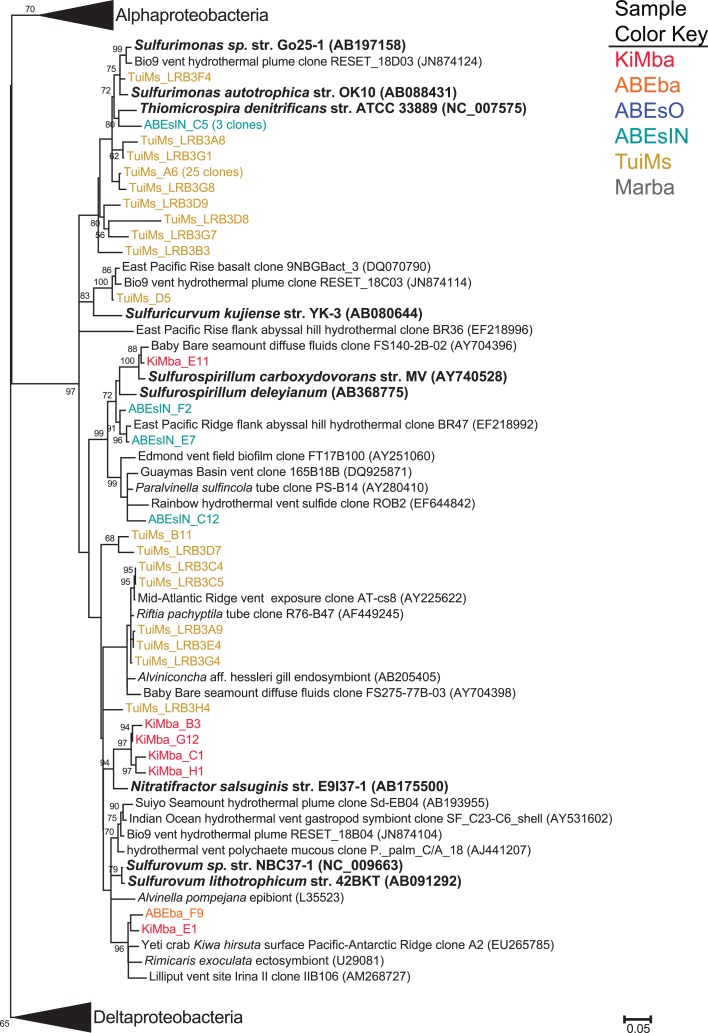
**Phylogenetic tree of all representative clones from this study that fall within the ε-Proteobacteria**. The tree was generated in MEGA 5 (Tamura et al., 2011) using the maximum likelihood method with the Jukes–Cantor model and a Gamma distribution and 500 bootstrap replicates. Nodes where bootstrap support is >50% are indicated. Samples are color-coded and no representative clones are shared across multiple samples.

Many of the bacterial clones recovered from silicate samples KiMba and ABEba are closely related to isolates involved in sulfur, methane, and hydrogen biogeochemical cycling. Within the γ-proteobacteria, this includes clones that fall within the order Methylococcales, aerobic methanotrophs, as well as OTUs allied to the orders Thiotrichales and Chromatiales (Figure 8). Known sulfur oxidizers populate both of these orders. Clones recovered from the basalts classified as ε-proteobacteria are most closely related to the genera *Sulfurospirillum*, *Nitratifractor*, and endosymbionts of vent macrofauna (Figure A3 in Appendix). Species within the genus *Sulfurospirillum* heterotrophically oxidize sulfur while those within *Nitratifractor* chemoautotrophically couple nitrate reduction with H_2_ oxidation (Nakagawa et al., 2005; Campbell et al., 2006). The phylum δ-proteobacteria is represented by clones from all three silicate samples, indicating that sulfur oxidation and reduction are likely occurring within different niches on the same rocks.

Previous studies have also noted the co-occurrence of sulfur oxidizing and sulfur reducing bacteria on seafloor-exposed basalts (Santelli et al., 2009; Sudek et al., 2009). This indicates that pores within the rocks provide different niches for diverse microbial lifestyles. Fresh basalt is rich in reduced sulfur and therefore sulfur oxidizing lineages are expected (Bach and Edwards, 2003). However, sulfur reducers (both sulfate reducers and bacteria carrying out sulfur disproportionation) may thrive in anaerobic and microaerophilic pockets of these same rocks, where they respire seawater sulfate or oxidized sulfur compounds in the rock substrate, such as elemental sulfur. This is known to occur with iron-respiring microbes on basalts – iron-oxidizers and anaerobic iron-reducers can be grown on separate incubations of the same basalt rock (Bailey et al., 2009), and therefore, could occur with sulfur respiring bacteria as well.

### Inactive sulfides

We observed differences between the bacterial communities detected on silicate samples and those detected on the inactive sulfides. This is in agreement with recent work that shows geochemistry strongly influences bacterial community membership on a given substrate, even when temperature and location are similar (Toner et al., 2013), as is the case here for samples ABEba, ABEsO, and ABEsIN, which were all collected from ABE vent field. Further, we found significant differences between bacterial communities detected on the outer chimney wall and inner conduit of the same inactive sulfide chimney, ABEs. Similar zonation was noted in prior studies of both active and inactive sulfide chimneys (Schrenk et al., 2003; Suzuki et al., 2004; Kormas et al., 2006; Sylvan et al., 2012a), likely due to the diverse chemical microenvironments that exist in chimneys, providing many niches for microbes (Kristall et al., 2011).

Sample ABEsO is host to a population of γ-proteobacteria that belongs to the SUP05 clade (Figure 5). The SUP05 clade was originally detected in the hydrothermal plume of the Suiyo Seamount (Sunamura et al., 2004) and has since been detected in hydrothermal plumes in vent fields from Guaymas Basin (Dick and Tebo, 2010), the Mid-Cayman Rise (German et al., 2010), and globally distributed oxygen minimum zones (Walsh et al., 2009). A single clone was also detected on an inactive sulfide collected from the Okinawa Trough (Kato et al., 2010). Metagenomic analysis indicates that these organisms are autotrophic sulfur oxidizers (Walsh et al., 2009). The SUP05 related clones detected in sample ABEsO indicate that these organisms are largely responsible for sulfur oxidation on the outside of this sulfide chimney, but this clade was not detected on the inside of the same sample (ABEsIN). There, a clade that falls within the Chromatiales order, represented by cloneABEsIN_A4 and three nearby clones (Figure 5), as well as a few ε-proteobacterial clones, represented by clone ABEsIN_C5 (Figure 6), likely fulfill the role of sulfur oxidation. These differences are likely due to affinity for different mineralogy between these two clades, or residual community differences inherited from past temperature and geochemical regimes within the active structure.

Clones TuiMs_LRB3C7, TuiMs_LRB3F6, TuiMs_C8, and TuiMs_B9 (Figure 6) all belong to the α-proteobacterial family Rhodobacteraceae and are most similar to cultured representatives belonging to the genera *Sulfitobacter* and *Roseovarius*. All the species from these genera whose genome has been sequenced contain the *sox* cluster of genes that imparts the ability to oxidize sulfur (Newton et al., 2010). Therefore, it is likely that inactive sulfide TuiMs harbors at least three different niches for sulfur oxidation to accommodate for S-oxidizing SUP05 bacteria, ε-proteobacteria, and Rhodobacteraceae.

The clones representative of δ-proteobacterial lineages recovered from ABEsIN are only distantly related to their closest cultured relatives; clone ABEsIN_D9 is 83% similar to *Nitrospina griacilis*, a nitrite oxidizer, and clone ABEsIN_A1 is 89% similar to both Mono Lake isolate strain MLMS-1 and *Desulfovibrio alkaliphilus* str. AHT2. This former isolate autotrophically couples sulfur oxidation with As-reduction (Hoeft et al., 2004), while the latter oxidizes reduced sulfur compounds (Sorokin et al., 2008). Therefore, it is impossible to assign an ecological role to these clones. As on the silicates studied here, there are also clones representative of δ-proteobacteria that are most likely representative of sulfur reducers on the same rocks with sulfur oxidizers (e.g., TuiMs_B8; Figure 6). This indicates multiple niches within these sulfides, as mentioned above.

**Figure 9:**
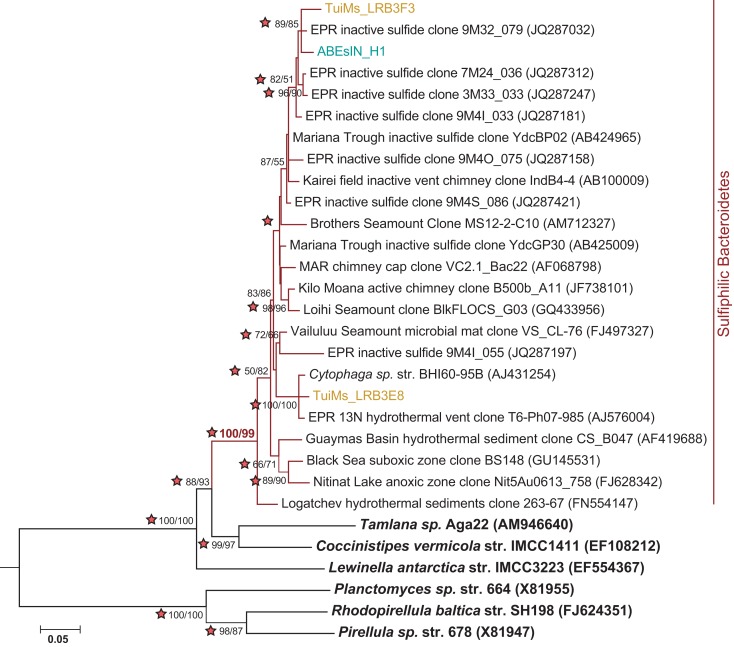
**Phylogenetic tree of representative clones from this study that fall within the Sulfiphilic Bacteroidetes clade (red branches)**. One representative clone was chosen from each study and from each sample if multiple samples are represented from a study. The tree was generated in MEGA 5 (Tamura et al., 2011) using the maximum likelihood method with the Jukes–Cantor model, Gamma distribution and 1000 bootstrap replicates. The same alignment was used to generate a neighbor-joining tree with the maximum composite likelihood method and 1000 bootstrap replicates. Nodes where both methods agree and bootstrap support is >50% are indicated with bootstrap values from the neighbor-joining tree on the left and the maximum likelihood tree on the right. Stars indicate nodes supported by Bayesian analysis. *Nitrospira marina* was used as an outgroup.

Clones TuiMs_LRB3F3 and ABEsIN_H1 (Figure 7) group with closely related clones recovered from inactive sulfides collected from the EPR (Sylvan et al., 2012b) and Indian Ocean (Suzuki et al., 2004). In the EPR study, these clones represented 17% of all the clones recovered, and were detected on six of seven samples. Similar clones detected *via* BlastN (Altschul et al., 1990) in the NCBI database are all from inactive sulfides collected during these previous studies or sulfidic environments such as active hydrothermal vents, oxygen minimum zones, and sediments. Phylogenetic analysis indicates the Bacteroidetes represented by these clones form their own clade (Figure 9), for which we propose the name of “Sulfiphilic Bacteroidetes.” This group includes clones recovered exclusively from sulfidic environments, but no published isolates currently exist. The Sulfiphilic Bacteroidetes fall within the Bacteroidales order of the Bacteroidetes; at this broad level of classification, it is impossible to assign an ecological niche to the organisms represented by these clones. It is, however, likely that the Sulfiphilic Bacteroidetes require reduced sulfur for growth, given that they are recovered exclusively from sulfidic environments.

Following cessation of venting on a sulfide chimney, a different bacterial assemblage is known to succeed the one present during active venting (Sylvan et al., 2012a). This assemblage is unique from that found on active chimneys (Kato et al., 2010; Sylvan et al., 2012a) and is also different from bacterial communities on other geological substrates in the deep ocean, such as seafloor basalts and sediment (Toner et al., 2013). One of the hallmarks of the succession on hydrothermal sulfide structures is the much lower proportion of ε-proteobacteria on inactive sulfides (Sylvan et al., 2012a); however, ε-proteobacteria in the genera *Sulfurimonas* (81% of ε-proteobacteria on TuiMs) and *Sulfurovum* (9%) represented a surprisingly high proportion on inactive sulfide sample TuiMs. The bacterial community on this sample was most similar to that on two inactive sulfides collected from the EPR, also with higher percentages of ε-proteobacteria than other inactive sulfides (Figure 8). This may indicate that these samples are only recently inactive and calls for dating of inactive sulfide samples in the future. The presence of anhydrite on sample TuiMs supports this argument – anhydrite is a known component of active chimneys but less common on inactive sulfides (Haymon and Kastner, 1981), and it is a major component of active sulfides at the nearby Mariner hydrothermal vent field (Takai et al., 2008). Nearby active chimneys at Tui Malila were dominated by ε-proteobacteria in the genus *Lebetimonas* and had nearly no *Sulfurimonas* (Flores et al., 2012), therefore it is possible that the bacterial community on chimney TuiMs was in a transition from a thermophilic community similar to those found on active vents at the Tui Malila vent field to the community we detected, which is more indicative of mesophilic microbes. The absence of detectable Archaea on TuiMs also supports the transition away from a microbial community representative of an actively venting sulfide.

The mineralogy of inactive sulfides appears to influence the composition of the extant bacterial communities (Kato et al., 2010; Toner et al., 2013). Indeed, our analysis reveals that there is no biogeographical pattern amongst bacterial communities on inactive sulfides; samples from the Mariana Trough and EPR fall on different branches of Figure 8, indicating that populations from the same ocean basin are not always most similarly related to each other. Closer inspection reveals that the composition of bacterial communities is heterogeneous and potentially unique on each structure. In every case where more than one sample was collected from a single sulfide structure (9M4 from the EPR, Kair from the Indian Ocean Ridge, Pltc, and Ydc from the Mariana Trough, and Ihe from the Okinawa Trough), all samples from that sulfide group together on the same branch. The sole exception to this rule is sulfide ABEs, for which the inside conduit harbors a dramatically different bacterial community than the outside wall. This indicates that there is much heterogeneity from one inactive sulfide structure to another, and we still have much to learn about these inactive sulfide ecosystems. Future studies can learn more about the potentially diverse microenvironments within these structures by finely sampling for both mineralogy and microbiology.

### Implications for weathering of seafloor rocks

The noted patterns in microbial communities, both from north to south and between different substrates, have implications for weathering of seafloor rocks. It is known that bacteria incubated with basalt enhance Si, Fe, and Mn release into the aqueous phase (Daughney et al., 2004; Edwards et al., 2004), and it is also likely that differences in substrate composition, which is a known driver of microbial composition (Toner et al., 2013), drive differential weathering rates and products released by endolithic microbes. Microbial biofilms on seafloor incubated hydrothermal sulfides indicate the presence of iron oxyhydroxides (Toner et al., 2009) and a preference by microbes for minerals that are both highly soluble and porous (Edwards et al., 2003). All of these processes interact and result in both bioalteration of seafloor rocks and release of weathering materials into the water column. This is poorly quantified but likely important for ocean biogeochemistry. It should be noted out that in addition to Bacteria and Archaea, which were analyzed here, there is growing evidence that fungi are also likely important in weathering processes in deep-sea environments (Biddle et al., 2005; Lopez-Garcia et al., 2007; Smith et al., 2011; Ivarsson et al., 2012).

## Conclusion

It is likely that the north-south gradients in bacterial community composition are driven by the differences in substrate chemistry between these fields. Many elements display linear transitions from Kilo Moana to Mariner, which makes it difficult to pinpoint which one (or ones) may be driving the observed differences in microbiology, but we were able to detect correlation between the elemental composition of the collected samples and bacterial phyla Nitrospirae and Chloroflexi (Table 4). Additionally, with the small sample size of this study, it is important to point out that the patterns observed here warrant further investigation and would benefit from additional and more intensive sampling. The samples collected for this study represent an initial foray into the microbiology of low temperature deposits in Lau Basin.

Bacterial communities detected on low temperature silicates and inactive sulfides along the ELSC and VFR in Lau Basin display distinct patterns that are driven by (1) gradients in rock geochemistry from north to south on the silicates, and (2) differences in substrate between the basalts, basaltic andesite, and inactive sulfides. The prevalence of Chloroflexi clones distantly related to any known isolates on basaltic andesite indicates that this substrate may host unique microbial populations. Clones related to organisms involved in multiple facets of sulfur and iron oxidation-reduction processes on the same rocks indicate that multiple micro-niches are present on both silicates and sulfides. Further, multiple niches for the same ecological function, like sulfur oxidation by three different clades of organisms on sulfide ABEs, indicate that fine scale differences in mineralogy likely support similarly nuanced microbial communities. Bacterial communities on inactive sulfides from Lau Basin are not unlike others studied at different sites, but it appears that each structure studied harbors a bacterial community most similar to itself and, therefore, further study of these ecosystems with finer resolution is warranted.

## Conflict of Interest Statement

The authors declare that the research was conducted in the absence of any commercial or financial relationships that could be construed as a potential conflict of interest.
